# Experimental Evolution of *Legionella pneumophila* in Mouse Macrophages Leads to Strains with Altered Determinants of Environmental Survival

**DOI:** 10.1371/journal.ppat.1002731

**Published:** 2012-05-31

**Authors:** Alexander W. Ensminger, Yosuf Yassin, Alexander Miron, Ralph R. Isberg

**Affiliations:** 1 Howard Hughes Medical Institute, Boston, Massachusetts, United States of America; 2 Department of Molecular Biology and Microbiology, Tufts University School of Medicine, Boston, Massachusetts, United States of America; 3 Department of Molecular Genetics, University of Toronto, Toronto, Ontario, Canada; 4 Public Health Ontario, Toronto, Ontario, Canada; 5 Department of Cancer Biology, Dana Farber Cancer Institute, Boston, Massachusetts, United States of America; Yale University School of Medicine, United States of America

## Abstract

The Gram-negative bacterium, *Legionella pneumophila*, is a protozoan parasite and accidental intracellular pathogen of humans. We propose a model in which cycling through multiple protozoan hosts in the environment holds *L. pneumophila* in a state of evolutionary stasis as a broad host-range pathogen. Using an experimental evolution approach, we tested this hypothesis by restricting *L. pneumophila* to growth within mouse macrophages for hundreds of generations. Whole-genome resequencing and high-throughput genotyping identified several parallel adaptive mutations and population dynamics that led to improved replication within macrophages. Based on these results, we provide a detailed view of the population dynamics of an experimentally evolving bacterial population, punctuated by frequent instances of transient clonal interference and selective sweeps. Non-synonymous point mutations in the flagellar regulator, *fleN*, resulted in increased uptake and broadly increased replication in both macrophages and amoebae. Mutations in multiple steps of the lysine biosynthesis pathway were also independently isolated, resulting in lysine auxotrophy and reduced replication in amoebae. These results demonstrate that under laboratory conditions, host restriction is sufficient to rapidly modify *L. pneumophila* fitness and host range. We hypothesize that, in the environment, host cycling prevents *L. pneumophila* host-specialization by maintaining pathways that are deleterious for growth in macrophages and other hosts.

## Author Summary

*Legionella pneumophila* is an accidental pathogen of humans, responsible for the severe, often-fatal pneumonia known as Legionnaires' disease. In the environment, *L. pneumophila* survives and replicates within protozoa by co-opting the intracellular machinery of these microbial predators. These freshwater encounters between bacteria and protozoa likely provided *L. pneumophila* with the selective pressures required to evolve into an intracellular pathogen. Many of the host pathways that *L. pneumophila* manipulates during infection are highly conserved and this is presumably what allows *L. pneumophila* to infect human cells. It is likely that *L. pneumophila* is suboptimally adapted to replication within mammalian cells, however, as replication within human cells is thought to be an evolutionary dead end. In this study, we developed an experimental evolution approach to determine what unique selective pressures might be present within mammalian hosts and how these pressures might modify this pathogen. We subjected *L. pneumophila* to continuous passage within mouse macrophages for several months, selecting for spontaneous mutations that resulted in improved fitness within these cells. We sequenced the genomes of each of the adapted strains, measured the population dynamics of each evolving population, and identified mutations that improve replication in mammalian cells and alter bacterial fitness in amoebae.

## Introduction

*L. pneumophila* is a Gram-negative intracellular pathogen with a broad host range that extends from unicellular protozoa to alveolar macrophages of the human lung [1]. *L. pneumophila* is an accidental pathogen: responsible for severe, sporadic disease in humans [2]–[4], but ubiquitous in nature [5]–[7]. Natural and man-made freshwater sources serve as the primary environmental reservoirs of *L. pneumophila*, with bacterial replication occurring within a diverse set of protozoan species within the aquatic environment. *L. pneumophila* has been shown to replicate in over 15 species of protozoa [8]–[10], consistent with the bacterium being a generalist in that it shows little evidence of species specificity. After uptake by these natural protozoan hosts, the *L. pneumophila* type IVB Dot/Icm translocation system translocates a large cadre of proteins across host membranes [11]–[13], remodeling the *Legionella*-containing vacuole (LCV) into a non-acidified compartment supportive of intracellular replication [14]–[16]. Over 300 bacterial proteins are thought to be substrates of this translocation system [6], [7]. Recent evidence supports a model in which the large repertoire of Dot/Icm translocated substrates is essential to the broad host range of *L. pneumophila*, with different subsets of these proteins contributing to optimal replication in distinct protozoan hosts [17]. We hypothesize that host cycling in the environment maintains *L. pneumophila* as a generalist, presumably through purifying selection against mutations that diminish fitness in any of several naturally encountered protozoan hosts.

Environmental replication of *L. pneumophila* within man-made water sources frequently leads to human exposure to the bacteria, through inhalation of contaminated aerosols [18]. *L. pneumophila* is the causative agent of Legionnaires' disease, a severe, often-fatal pneumonia [19] and Pontiac Fever, a less severe, self-limiting disease [20]. Once inside the human lung, *L. pneumophila* bacteria are able to replicate within alveolar macrophages in a process that appears broadly similar to that which occurs within amoebae in the natural environment [21]. As in amoebae, the establishment by *L. pneumophila* of a non-acidified, replicative vacuole in macrophages is critically dependent on components of the Dot/Icm translocation system [22], [23].

During the evolutionary history of *L. pneumophila*, encounters between these bacteria and mammalian host cells are likely to be quite rare relative to their persistent encounters with protozoan hosts. No environmental mammalian reservoirs of *L. pneumophila* have been identified and, while *L. pneumophila* bacteria are capable of causing severe disease within humans, there is no evidence of human-to-human transmission of the bacteria [24]. For instance, when infected individuals returned home from the 1976 Philadelphia outbreak of Legionnaires' disease, none of the 193 surveyed contacts of these individuals developed symptoms of the disease [24]. This suggests that the interaction between *L. pneumophila* and mammalian host cells is likely an infrequent event of limited duration and may represent an evolutionary dead-end from the perspective of the pathogen. Therefore, during outbreaks of human disease, it is likely that the macrophages encountered by *L. pneumophila* represent a novel host environment for which each inoculating bacterium is suboptimally adapted.

While many similarities exist between the shared intracellular survival strategies employed by *L. pneumophila* in both amoebal and mammalian host cells, little is known about the differences between these two intracellular environments. The adaptation of *L. pneumophila* to the intracellular niche is thought to have occurred within a diverse range of protozoan hosts in the natural environment [8]–[10], resulting in a pathogen with a remarkably broad host range. Conservation between protozoa and humans of several *L. pneumophila* targets, such as key host components of intracellular vesicular trafficking [25], is presumably responsible for the accidental pathogenesis of *L. pneumophila* in human hosts [9]. The same selective pressures are thought to have selected for a pathogen exhibiting significant genetic redundancy [17], making *L. pneumophila* recalcitrant to many forward genetic screens aimed at uncovering the function of individual virulence factors [26], [27].

We sought to directly test whether unique selective pressures are placed upon bacteria replicating within mammalian cells by restricting *L. pneumophila* to growth within mouse macrophages for hundreds of generations. Multiple mutations were identified that improved fitness in macrophages, either in isolation or synergistically with other mutations. Many of these mutations altered bacterial replication in natural amoebal hosts, suggesting fitness trade-offs between natural and accidental hosts. These data represent the first directed adaptation of *L. pneumophila* host range, a powerful experimental approach to understanding the evolution of host-pathogen interactions within specific host cell-types.

## Results

### Experimental evolution of *Legionella pneumophila* leads to strains with improved replication in mouse macrophages

**Figure 1:**
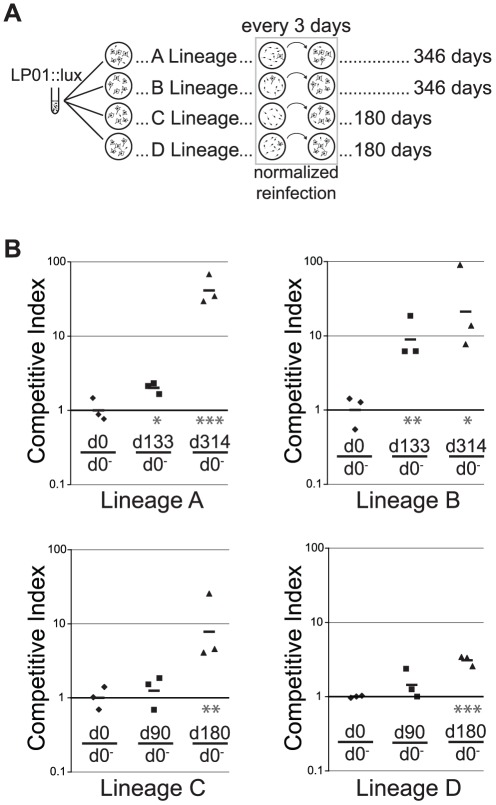
Experimental evolution of *Legionella pneumophila* leads to strains with improved replication in mouse macrophages. (**A**) Strategy for extended passage of *L. pneumophila* through primary A/J bone marrow-derived macrophages. *L. pneumophila ahpC^+^/ahpC::luxCDABE* was cultured overnight and inoculated into primary bone marrow-derived macrophages from A/J mice at MOI = 0.05 (Materials and Methods). After 3 days in culture, bacteria were harvested and used to re-infect new macrophages, using luminescence to maintain consistent MOIs in subsequent passages. (**B**) Passaged isolates have a competitive advantage relative to the progenitor strain in A/J macrophages. Clonal isolates from each lineage, as well as the luminescent progenitor (d0), were co-inoculated with a non-luminescent strain (d0^−^) into A/J macrophages. (The genotype of each clone is listed in Table S1). Total bacteria from the inoculum and from 3 days post-inoculation were plated onto non-selective media and imaged with and without white epi-illumination (Materials and Methods). d0 = progenitor strain; d133 = single colony isolate after passage for 133 days in/J macrophages. Competitive index (C.I.) = ratio output/ratio input, normalized to d0/d0^−^. Each bar represents the geometric mean of data from 3 independent infections (points). An unpaired, two-tailed Student's t-test was performed on each logarithmic transformed C.I. relative to that of the d0/d0^−^ control: *: p<0.05; **: p<0.01; ***: p<0.001.

*L. pneumophila* Philadelphia-1, strain LP01 [22], was modified to contain an integrated *lux* operon [28] and used to inoculate four independent cultures of 1×10^7^ primary A/J bone marrow-derived mouse macrophages at a multiplicity of infection (MOI) of 0.05 bacteria per host cell (Figure 1A). Under these culture conditions, *L. pneumophila* was unable to replicate outside of host cells (data not shown), but could replicate intracellularly for approximately 3 days before exhaustion of the macrophage culture. After 2–3 days, any remaining host cells were lysed, bacterial numbers were estimated using luminescence, diluted, and used to inoculate new macrophages, again at a low MOI (estimated 0.05, see Materials and Methods). Samples of viable bacteria were taken every 10–20 days to allow for the study of intermediate time-points. In this manner, four independent lineages of *L. pneumophila* were confined to intracellular replication within mouse macrophages for several months.

To determine if extended replication within macrophages would improve the fitness of *L. pneumophila* within this environment, competition experiments were performed [29]–[31] (Figure 1B). Macrophages were incubated for 3 days with both non-luminescent *L. pneumophila* and clones isolated from each of the four lineages at various points during passage. Bacteria were then plated on solid agar and the ratios of luminescent to non-luminescent colony forming units were determined through imaging. After each of these competitions, the relative frequency of the adapted strains was greater than that of the progenitor. Within each lineage, clonal isolates from later time-points uniformly displayed greater growth advantages than did those from earlier time-points (Figure 1B).

### Population dynamics of mutations that emerge during intracellular passage

We next identified the mutations that arose during passage in macrophages. Clones from each lineage were used to generate libraries for whole-genome sequencing (Materials and Methods). Reference and *de novo* genome assembly software [32] was used to identify differences between the passaged and progenitor strains (Table S1). These analyses identified several point mutations, small insertions, small deletions, and the precise start and stop points of one large (45.5 kb) deletion known to exist in the LP01 laboratory strain [33]. Unlike other *in vitro* bacterial evolution studies [34], we did not observe differences in insertion sequence number or location, though it is possible that the strategies we used were not optimal for detecting alterations in insertion sequence copy numbers (see Materials and Methods). Several independent mutations in the flagellar regulator, *fleN* [35], [36], components of the lysine biosynthesis pathway, and the Dot/Icm translocated substrate, *sdbA* [37], were identified. Two clones from independent lineages contained an identical single nucleotide deletion, consistent with a mutational hotspot between *pacS*, a putative cation transporter, and *lphB*, a gene adjacent to several Dot/Icm components [23]. Of the 27 mutations identified across all lineages, 5 are predicted to result in frameshifts in open reading frames, 4 are nonsense mutations, 13 are non-synonymous missense mutations, 3 are intergenic, and 2 are predicted to be synonymous.

**Figure 2:**
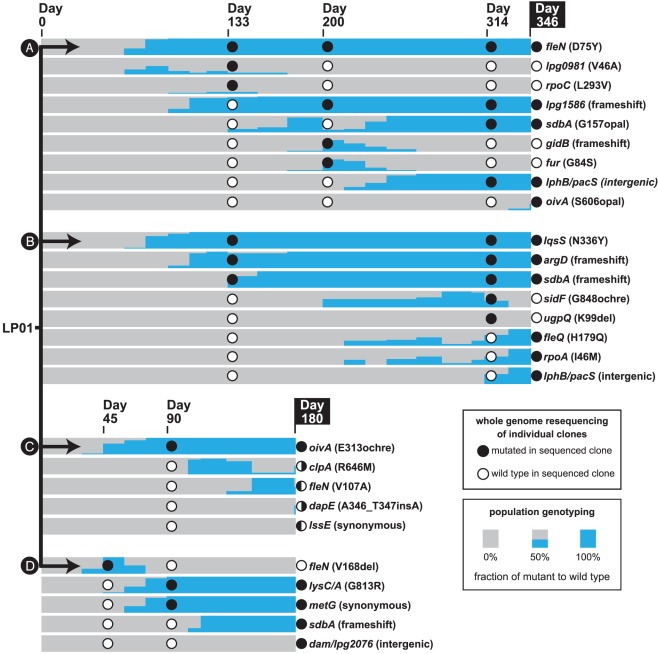
Fixation of mutations and clonal interference during long-term passage in macrophages. Whole genome sequencing using an Illumina Genome Analyzer II was used to identify individual mutations in several clones isolated from each lineage (**A–D**) of macrophage-adapted *L. pneumophila*. The presence of a mutation in a sequenced clone is indicated by a solid circle at that time-point, with the genotype of a single clone represented as a column of such circles. The effect of each mutation on each corresponding protein is indicated in parentheses. Split circles indicate that multiple clones were sequenced from the same time, with discrete genotypes. Population genotyping was performed on uncultured glycerol stocks, using high-throughput qEGAN analysis (see Materials and Methods), to determine the prevalence of each mutation over time.

To quantify the frequency of each mutation across the duration of passage, we next used population genotyping (qEGAN analysis [38], Materials and Methods) to compare populations from each intermediate time-point to known mixtures of wild type and mutant genomic template (Figure 2). By providing an unprecedented level of genotypic detail across the entire duration of extended passage, these data uncovered population dynamics consistent with selective sweeps, such as *fleN*(D75Y) becoming fixed in lineage A by day 118. In addition, there were several instances of clonal interference, in which subpopulations transiently increased in frequency. For example in lineage D, a strain carrying the *fleN*(V168del), which was the predominant clone at day 45, was ultimately replaced by bacteria harboring the *lysC/A metG* double mutation.

This analysis also uncovered population changes that were not readily explained by the mutations identified in the initial set of clones chosen for sequencing. The proportion of mutants in lineage A having the *sdbA*(G157opal) lesion increased from days 133 to 175, but reduced dramatically at day 200 (Figure 2). The frequency of *sdbA*(G157opal) in the population then increased to apparent fixation concurrently with the acquisition of a mutation between *lphB* and *pacS*. As this population behavior was consistent with transient clonal interference from a subpopulation of bacteria, we sequenced a *sdbA^+^* strain isolated from day 200 for further analysis, and identified two additional mutations, one in *gidB* and the other in what is annotated as an intergenic mutation between *smpA* and *cdgS7* (Table S1, Figure 2). We performed TBLAST analysis [39] on the *smpA* and *cdgS7* intergenic region, and identified a conserved intact open reading frame with an alternate GTG start codon corresponding to the ferric uptake regulation (*fur*) gene [40]. This open reading frame is missing from the published *L. pneumophila* Philadelphia-1 genome annotation [41], perhaps due to its alternate GTG start codon. As predicted, subsequent population genotyping analysis demonstrated that the frequency of the *gidB* and *fur* mutations in lineage A was inversely correlated to the frequency of *sdbA* from day 175 to 245. Using a similar approach, we identified *clpA* and *dapE* mutations in the C lineage from targeted resequencing of a *fleN^+^* isolate at day 180 (Table S1, Figure 2). The population dynamics observed after these iterative rounds of sequencing is consistent with the majority of the significant mutations within each lineage being identified by this approach.

### Extended passage in macrophages results in mutations in a flagellar regulator and increased uptake into host cells

**Figure 3:**
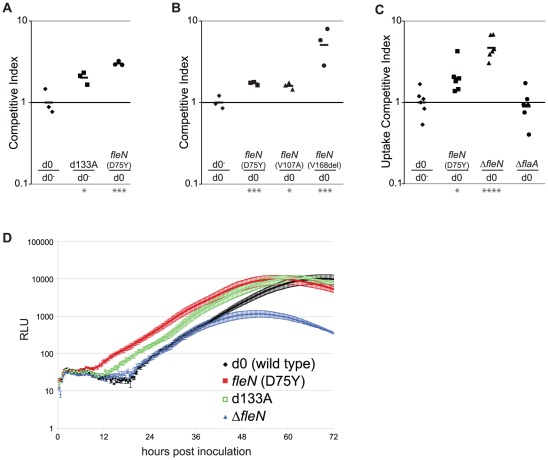
Mutations from clones that survived passage in macrophages allow outcompetition with the progenitor strain. (**A,B**) Competitions were performed between luminescent and non-luminescent strains by co-inoculating A/J macrophages with two different strains. Ratios were normalized to a luminescent/non-luminescent wild type progenitor (d0/d0^−^) control. The ratio of strains was determined by plating the inoculum and lysates taken 3 days post-inoculation. Difference from d0/d0^−^ as tested by an unpaired Student's t-test of log-transformed values: *: p<0.05; **: p<0.01; ***: p<0.001; ****: p<0.0001. (**A**) One of the mutations, *fleN*(D75Y), from the sequenced day 133A clone was introduced into the progenitor strain. Macrophages were co-inoculated with a wild type strain and either the passaged strain or *fleN*(D75Y). (**B**) Strains harboring *fleN* missense mutations outcompete wild type. Each of the three *fleN* mutations identified in the adapted strains were individually introduced into the progenitor background and co-inoculated into macrophages with the luminescent progenitor. (**C**) Both the *fleN(D75Y)* mutation and an in-frame *fleN* deletion (Δ*fleN*) cause enhanced uptake relative to wild type. Macrophages were co-inoculated with the progenitor strain, d0, and either *fleN*(D75Y), Δ*fleN*, or a Δ*flaA* strain lacking flagellin. After 1 hr of incubation, the cells were incubated with gentamicin for 1 hr, washed extensively and lysed. The ratio of strains was determined by plating the inoculum and lysates. (**D**) The in-frame deletion of *fleN* does not recapitulate the growth advantage of a spontaneous *fleN* point mutant selected for during passage in macrophages. Intracellular growth of the sequenced day 133A clone, *fleN*(D75Y), and Δ*fleN* strains in primary A/J bone marrow-derived macrophages. Host cells were inoculated with *L. pneumophila* at MOI = 0.05 in 96 well plates. In multiple independent experiments, cultures were incubated at 37°C, 5% CO2 in a Tecan M200 Pro plate-reader and luminescence of each well was measured every 20 minutes. The data are plotted as the average of 3 or more replicates of each strain at each time-point from a representative experiment. Error bars represent the standard error of the mean.

Mutations in *fleN* were identified in three independent lineages, suggesting that modulation of this locus might confer a growth advantage in macrophages. The *fleN* locus is located in a dense neighborhood of flagellar regulation genes in *L. pneumophila*, which may restrict the types of mutations that might otherwise be isolated from this locus. We introduced the *fleN*(D75Y) mutation into wild type bacteria and measured the fitness of this strain in macrophages through competition against the luminescent progenitor strain (Figure 3A). The introduction of this single amino-acid change was enough to recapitulate the competitive advantage of an A lineage clone isolated at 133 days. Other *fleN* mutations (identified in the C and D lineages) when placed into the wild type background conferred similar growth advantages (Figure 3B).

FleN is known to regulate flagellar number in other bacteria [36], [42], but little is known about its function in *L. pneumophila*. While both the *fleN*(D75Y) mutant and an in-frame deletion strain were motile and maintained a single polar flagellum by transmission electron microscopy (data not shown), we investigated whether known consequences of altered flagellar function could be observed. *L. pneumophila* with misregulated flagellar assembly often show lowered association with host cells [35], so we determined if the *fleN* non-synonymous mutations resulted in modified cell association. Macrophages were inoculated with equal ratios of luminescent and non-luminescent strains for one hour, incubated with gentamicin for one additional hour to kill extracellular bacteria, and then output to input ratios between strains were compared (Figure 3C). In these assays, there was enhanced internalization by macrophages of the *fleN*(D75Y) strain compared to wild type one hour after infection.

To determine whether these *fleN* non-synonymous point mutations phenocopied a complete loss-of-function mutation, we next constructed an in-frame deletion of *fleN* in the wild-type LP01 background. In uptake assays, the Δ*fleN* strain, like *fleN*(D75Y), showed improved uptake into primary mouse macrophages 1 hour after infection (Figure 3C). We next measured intracellular replication of wild-type LP01, the *fleN*(D75Y) point mutant, and two independently-derived Δ*fleN* strain, each carrying an integrated *lux* operon. Macrophages were challenged with each strain and luminescence was monitored over 72 hours of incubation to determine the growth dynamics of each strain. The *fleN*(D75Y) non-synonymous point mutant showed improved growth relative to the wild-type strain (Figure 3D). In contrast, the growth of the Δ*fleN* clone was indistinguishable or slightly reduced from wild-type in these hosts (Figure 3D). Two independently-derived Δ*fleN* clones displayed phenotypes that were indistinguishable from one another (data not shown). Therefore, the specific *fleN* alleles isolated in this study were missense mutations that were selected because they resulted in proteins that had altered activities. These data are consistent with *fleN* serving multiple regulatory roles during infection, a hypothesis that is supported by recent data showing that *fleN* also influences cell division in *Campylobacter jejuni* [43].

### Multiple mutations have synergistic effects on fitness

**Figure 4:**
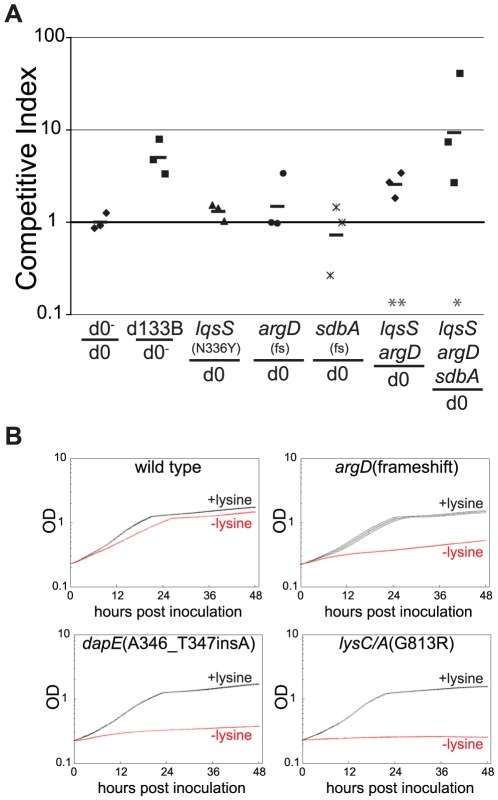
Mutations from clones that survived passage in macrophages can function synergistically and often result in strains displaying lysine auxotrophy. (**A**) Recapitulation of the B lineage phenotype by successive introduction of mutations in their order of becoming fixed. The *lqsS, argD*, and *sdbA* mutations, identified by sequencing a day 133 B lineage clone, were introduced into the progenitor strain in isolation and in combination. Competitions were performed by co-inoculating A/J mouse macrophages with each of these strains and the wild type progenitor. The ratios of strains were determined in both the inoculum and lysates taken 3 days later. (**B**) Mutations predicted to affect lysine biosynthesis pathway result in auxotrophy in broth culture. Each strain, having individual mutations introduced into the progenitor LP01 strain (noted in panels) was used to inoculate defined growth media with or without lysine and incubated at 37°C for 48 hours in a plate-reader. Absorbance at 600 nm (optical density, OD) was measured every 15 minutes. Data points represent the mean of 3 or more independent samples, error bars represent the standard error of the mean.

The mutations identified in lineage B were similarly analyzed by moving individual changes into the wild type parent. Multiple mutations were introduced into the LP01 parent both in isolation and in the order in which they emerged within the adapted population. The effect of these mutations was determined by competing these newly constructed strains against the LP01::*lux* strain (Figure 4A). In contrast to *fleN* mutations, multiple mutations were required to recapitulate the growth advantage of the lineage B isolate from day 133. Notably, the *lqsS*(N336Y), *argD*(frameshift), and *sdbA*(frameshift) mutations identified in lineage B did not detectably improve fitness in macrophages when placed in isolation, but introducing the *lqsS argD* (double) and *lqsS argD sdbA* (triple) mutations into LP01 resulted in improved fitness for both strains.

### Extended passage in macrophages results in lysine auxotrophy and reduced fitness in amoebae

Mutations were also observed within genes predicted to participate in the lysine biosynthesis pathway. To determine the impact of these mutations on the nutritional requirements of *L. pneumophila*, we introduced each mutation into LP01 and then measured the *in vitro* growth of each within defined broth media [44] in the presence or absence of added lysine (Figure 4B). Strains harboring the *argD*, *dapE*, and *lysC/A* mutations (from the B, C, and D lineages respectively), all grew in medium with lysine but, unlike LP01, were severely defective for growth in its absence. The addition of exogenous meso-diaminopimelic acid (meso-DAP), an intermediate metabolite directly converted into lysine by LysA in the last step of the pathway [45] partially rescued the growth of *argD* and *dapE* mutants in the absence of lysine, but failed to rescue the *lysC/A* mutant (data not shown).

**Figure 5:**
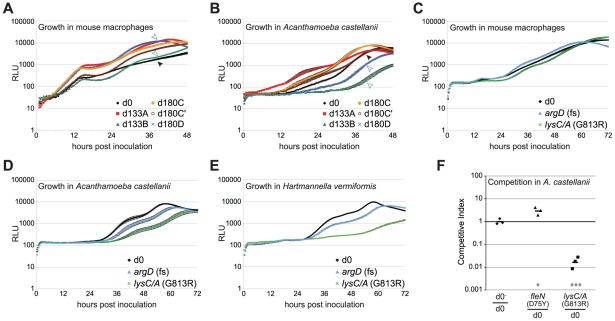
Single mutations selected during growth in macrophages impact growth in amoebae. Intracellular growth of macrophage-passaged *L. pneumophila* strains in (**A**) primary A/J bone marrow-derived macrophages and (**B**) *Acanthamoeba castellanii*. Host cells were inoculated with *L. pneumophila* at MOI = 0.05 in 96 well plates: d0 = progenitor; d133A = A lineage clone from 133 days; d133B = B lineage clone from 133 days; d180C = C lineage, clone from 180 days containing *fleN(V107A)*; d180C′ = C lineage, clone from 180 days containing *fleN^+^*; d180D = D lineage clone from 180 days. (The genotypes of each of these clones is indicated in Table S1.) Solid arrowhead (d0) and open arrowheads (d133B and d180D). In multiple independent experiments, cultures were incubated at 37°C, 5% CO_2_ in a Tecan M200 Pro plate-reader and luminescence of each well was measured every 20 minutes. The data are plotted as the average of 3 or more replicates of each strain at each time-point from a representative experiment. Error bars represent the standard error of the mean. (**C–E**) Individual *argD* and *lysC/A* mutations recapitulate the amoebal growth defects displayed by the B and D lineages from which they were initially isolated. (**F**) Competitions were performed by co-inoculating *A. castellanii* with the progenitor strain and strains harboring either a point mutation in *fleN* or the *lysC* region of *lysC/A* gene. Total bacteria from the inoculum and from 3 days post inoculation were plated onto non-selective media. Ratios of each strain were determined by imaging each plate with or without illumination and counting colony forming units under each condition. Competitive index = ratio output/ratio input, normalized to competitions between non-luminescent (d0^−^) and luminescent (d0) progenitor strains. Each bar represents the geometric mean of data from 3 independent infections (points). Difference from d0/d0^−^ as tested by an unpaired Student's t-test of log-transformed values: *: p<0.05, ***: p<0.001.

Models for the pathogenesis of *L. pneumophila* posit that the primary selective pressure for environmental maintenance of intracellular growth is the ability to replicate within amoebae [9], [46], [47]. Therefore, we tested if the adaptive changes affected growth in *Acanthamoeba castellanii*, one of several natural amoebal hosts of *L. pneumophila*, to determine if increased fitness in macrophages represents broadening of host range or causes a switch in host range specificity. As before, macrophages and amoebae were challenged with each strain and luminescence was monitored over 48–72 hours of incubation to determine the growth dynamics in both host cell types. The passaged strains replicated more efficiently in macrophages than did the progenitor strain (Figure 5A). In contrast, the lineage B and D strains displayed diminished growth in *A. castellanii* relative to the progenitor (Figure 5B). The two lysine biosynthesis mutations present in these strains, *argD* and *lysC/A*, were sufficient to generate these phenotypes in *A. castellanii* (Figure 5D) and in another amoebal host, *Hartmannella vermiformis* (Figure 5E). The *lysC/A*(G813R) also displayed a severe growth disadvantage during competitions with the wild type progenitor in *A. castellanii* (Figure 5F). The frequent observation of growth defects of lysine auxotrophs in *A. castellanii* and *H. vermiformis* is consistent with purifying selection during growth in environmental amoebae selecting for the maintenance of the wild type alleles of these genes, because the mutations that we analyzed resulted in a costly host-range specificity switch.

In general, the results of growth curves and competition experiments were highly reproducible. The one exception was the lineage C clone, d180C′, which contains a *dapE* mutation as well as two other mutations (Figure 2). We saw variable levels of growth defects for this strain during incubations with *A. castellanii* and *H. vermiformis*, indicating that this genotype selects for frequent suppressors or else this strain background is extremely sensitive to small variations in bacterial or host cell culture conditions.

In contrast to the lysine auxotrophs from the B and D lineage, the A and C lineage strains had equal or better fitness within amoeba as compared to the progenitor (Figure 5B). The A lineage mutation, *fleN*(D75Y), recapitulated this growth advantage in *A. castellanii* (Figure 5F), suggesting that some macrophage-driven adaptive changes can confer broadly increased replication in both macrophages and *A. castellanii*. None of the adapted strains displayed growth advantages during *in vitro* replication in rich media (data not shown), indicating that these phenotypic differences did not likely stem from the accumulation of mutations that broadly improved bacterial replication.

## Discussion

Analysis of bacterial experimental evolution has generally focused on identifying mutations that arise during growth in chemically defined media [48]–[52]. One of the signatures of the selected strains is that they contain mutations that give insight into critical features of the growth conditions used. For instance, continuous passage of *Escherichia coli* in minimal media containing glycerol as the sole carbon source results in selection of glycerol kinase mutations [49]. A parallel can be found in our studies with the isolation of lysine auxotrophs during bacterial passage in macrophages. This provides information about the metabolic requirements for growth in both the natural and mammalian host. First, this is consistent with lysine being delivered efficiently to the *Legionella*-containing vacuole (LCV) in mouse macrophages (Figure 2, Figure 4B). Secondly, as many of these auxotrophs grow worse in amoebae, the natural host appears to be inefficient at delivering lysine to the LCV (Figure 5D–F). Therefore, continuous passage represents a powerful approach that allows identification of the restrictive features of a poorly defined growth environment.

Experimental evolution of *L. pneumophila* within macrophages generated populations of mutants that had three features seen previously in experiments designed to model microbial adaptation in the laboratory as there was: 1) parallel evolution, with mutations being isolated in an overlapping set of loci across independent lineages; 2) frequent clonal interference between transient subpopulations of bacteria; and 3) genetic interactions, with the acquisition of successive mutations within a specific genetic background resulting in non-additive increases in the fitness levels of a strain.

The independent isolation of mutations in multiple parallel lineages that alter a single loci or biochemical pathway, known as parallel evolution, is a frequent result of experimental evolution enrichments [34], [49], [51], [53]–[55]. In multiple macrophage-adapted lineages of *L. pneumophila*, we also observed several instances of parallel evolution: non-synonymous mutations in the flagellar regulator, *fleN* in three lineages (with a *fleQ* mutation isolated in the fourth), nonsense mutations isolated in three lineages in the Dot/Icm translocated substrate, *sdbA*, three independent lysine biosynthesis mutations (*argD*, *dapE*, and *lysC/A*), nonsense mutations in 2-oxoisovalerate dehydrogenase (*oivA*) found in two lineages, and the same exact mutation in between a hypothetical protein, *lphB* and a putative copper transporter *pacS* in two lineages, suggesting a hotspot for this mutation.

By measuring the relative abundance of mutations in each population over time, all four of the macrophage-adapted lineages showed hallmarks of clonal interference, a property of asexual populations in which different beneficial mutations emerge in clones that subsequently compete with each other. Both experimental and mathematical modeling studies indicate that clonal interference is common in asexual populations and that these events should be positively correlated with both population size and mutation rate [56]–[58]. In our population genotyping data, we could readily identify time frames in which two mutations displayed inverse relative abundance, with only one reaching fixation in the population. In each case, we observed that the ultimate fixation of one of these mutations in a population was accompanied by the acquisition of one or more additional mutations. Our data cannot distinguish whether the acquisition of these additional mutations was stochastic or whether one of the competing genotypes was more or less compatible with additional beneficial mutations [59]. These two possibilities could be distinguished by replaying these events several times between the naturally competing clones of each lineage to determine whether one specific clone is more frequently fixed in each competition [59].

**Figure 6:**
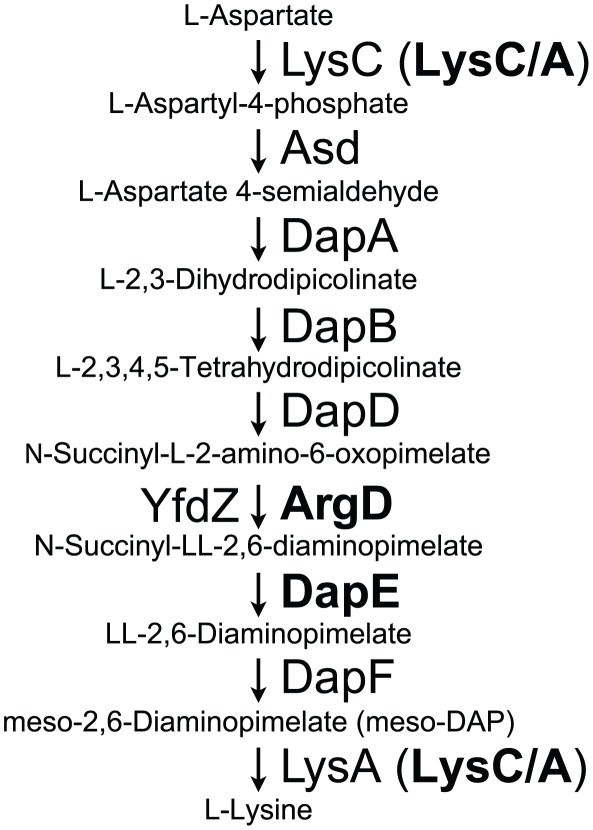
The lysine biosynthesis pathway in *L. pneumophila*. The *L. pneumophila* genome encodes for all of the proteins required for the biosynthesis of lysine from aspartate. Experimental evolution of *L. pneumophila* in mouse macrophages frequently led to lysine auxotrophic strains harboring mutations in either LysC/A, ArgD, or DapE (bold).

As organisms adapt to new environments, increased fitness under these conditions often correlates with reduced fitness in other environments [30], [60]. This relationship has been pursued using a number of model systems, including phage and other viruses adapted to novel host backgrounds [61]–[66], bacteria passaged under specific nutritional requirements [67], and light/dark cycling of the unicellular green algae, *Chlamydomonas* [68]. During extended passage through mouse macrophages, three out of four of the *L. pneumophila* lineages acquired mutations in lysine biosynthesis that resulted in lysine auxotrophy (Figure 2, Figure 4B). The most severe of these auxotrophic strains was the *lysC/A* mutation identified in lineage D. This gene encodes a bifunctional fusion protein predicted to catalyze both the first and last steps of lysine biosynthesis in *L. pneumophila* [69]–[72] (Figure 6), with the substitution occurring in the C-terminal domain that is similar to LysA, the DAP-decarboxylase responsible for converting meso-diaminopimelate (meso-DAP) to lysine. Consistent with this function, growth of this mutant in the absence of lysine cannot be rescued by addition of meso-DAP, whereas meso-DAP was able to partially rescue the growth of mutations in *argD* and *dapE* that are predicted to interfere with steps in the pathway upstream of meso-DAP (data not shown).

Selection for lysine biosynthesis defects could have arisen in two ways: 1) the macrophage may supply sufficient lysine to the replication vacuole, allowing accumulation of lysine biosynthesis mutations due to neutral selection for pathway maintenance; or 2) loss of lysine biosynthesis confers a fitness advantage within macrophages but with a corresponding fitness cost in other hosts [67]. Our data support the second model, known as antagonistic pleiotropy, because we observe parallel selection for these mutations in independent lineages (Figure 2), the acquisition of these mutations is rapid, and the fixation of each mutation appears to occur as part of selective sweeps through the population. If neutral selection were a significant contributor to the isolation of mutations in this study, we would expect to have identified lesions in several Dot/Icm translocated substrates that have individually and collectively been shown to be dispensable for growth in macrophages [1], [17]. The Dot/Icm translocated substrates represent over 10% of the entire *L. pneumophila* genome, yet the only mutations that we identified were in *sdbA* and *sidF*, which when mutated may provide a selective advantage [11]–[13]. As nutrient availability is known to regulate the intracellular differentiation of *L. pneumophila* from replicative to transmissive phase [73], we hypothesize that modulation of lysine biosynthesis may serve to influence the activation of transmissive traits during late stages of infection. Within this context, the potential for crosstalk between the lysine and arginine biosynthetic pathways in *L. pneumophila* should be explored given the inclusion of *argD* in both pathways and a previously identified role for arginine levels in broadly regulating transcription [74].

Our data are consistent with a model in which the availability of lysine (or intermediate metabolites in the lysine/DAP biosynthesis pathway) is lower in at least some amoebal hosts than in bone marrow-derived macrophages, which explains why this biosynthesis pathway has been retained in *L. pneumophila*. The macrophage-selected *lysC/A* (DAP-decarboxylase) and *argD* mutants are defective for intracellular growth in *A. castellanii* and *H. vermiformis* (Figure 5D–F), consistent with the model that available lysine pools are lower in some amoebae than in other cells. Based on genome annotation, *Coxiella burnetii* [75], an obligate mammalian pathogen and close relative of *L. pneumophila*, lacks a functional *lysA* DAP-decarboxylase, showing a connection between pathogen adaptation to mammalian host cells and selection for loss of a complete lysine biosynthesis pathway. *C. burnetii* survival, but not replication, has been demonstrated in amoebal hosts [76]. We propose that lysine availability within amoebae limits the environmental dissemination of mammalian host-adapted pathogens and prevents specialization of *L. pneumophila* for growth within a mammalian niche. A recent outbreak of Q-fever was linked to the presence of *C. burnetii* in an air-conditioning unit, where it was proposed that amoebal hosts supported persistence of the pathogen in the aquatic environment [77]. Strain-to-strain variation of the lysine biosynthesis pathway in these clinical strains may explain the dissemination of *C. burnetii* during this outbreak. In light of these results, a systematic examination of the nutritional requirements of *L. pneumophila* within diverse host backgrounds that was initiated several years ago should also be revisited in this new context [78].

In conclusion, we have shown rapid parallel evolution of *L. pneumophila* during conditions of host restriction in mouse macrophages. Some of the adaptations resulted in fitness costs in chemically defined media and in amoebal hosts, whereas others provide broadly improved intracellular replication. These data are consistent with the model that cycling through diverse protozoan hosts maintains *L. pneumophila* in “evolutionary stasis” as a generalist [17]. The extension of our experimental evolution approaches to conditions of host restriction in different protozoan species will be critical to determining whether genome reduction and/or specialization can occur under these conditions as well. *In vivo* experiments with West Nile Virus, indicate that having relaxed purifying selection within some hosts can also influence pathogen evolution during host cycling [61]. As additional natural protozoan hosts of *L. pneumophila* are identified, it will be important to determine whether similar processes also influence the evolution of *L. pneumophila* during its passage through diverse natural hosts.

Intracellular growth in isolated mouse macrophages can never capture the complexity of infection conditions in human hosts. While our adapted strains display improved replication in cultured mouse cells, both known deficits in the progenitor strain [79] and evolution towards reduced fitness in natural hosts would likely reduce their transmission in the wild. Remarkably little is understood about the transmission of *L. pneumophila* between its natural protozoan hosts and the human lung [80], the relationship between infectious dose and severity of disease, or genotypic diversity and selection during individual cases of disease. Just as access to low-cost sequencing has transformed the field of experimental evolution, the application of these technologies to environmental and clinical strains of bacteria has already started to define the selective pressures that influence the ultimate outcome of disease [81]–[86]. Applying these approaches to understanding the epidemiology of human outbreaks of *L. pneumophila* will be particularly critical to identifying the evolutionary pressures that shape these events.

## Materials and Methods

### Bacterial and host cell growth and transgenics

The *L. pneumophila* LP01 strain used in these studies is derived from a clinical isolate of *Legionella pneumophila* strain Philadelphia-1 [22] and is virulent in guinea pigs (data not shown). LP01, rather than a related thymidine auxotroph, LP02, was selected for these studies in order to facilitate amoebal challenge, as thymidine auxotrophy severely limits intracellular replication in these hosts. pSR47-*ahpC::lux*, a plasmid containing the *luxCDABE* operon of *Photorhabdus luminescens* downstream of the *L. pneumophila ahpC* promoter (a kind gift from J. Coers and R. Vance) [28], was used in triparental matings with the *E. coli* Tra^+^ helper strain RK600 [87] to integrate the *lux* operon onto the chromosome of *L. pneumophila* strain LP01. Individual mutations were introduced to LP01 through triparental matings with pSR47S-derived plasmids containing 2 kb PCR products generated from amplification of regions flanking identified mutations using genomic DNA of each passaged strain as template. An in-frame deletion of the *fleN* open reading frame in LP01 was generated by first making a pSR47S-derived plasmid containing 4.9 kb of sequence surrounding the *fleN* locus. Inverse PCR was then used to eliminate residues 27–284 of the 295 amino acid protein within this plasmid, using lpg1783ko_invF (5′-TCAAGGCAGATCTTTTCTTTTTGGAGCGTTTGG-3′) and lpg1783ko_invR (5′-TCAAGGCAGATCTCGGGACAAATTTCTAAGACCA-3′), followed by BglII/DpnI digestion and subsequent intra-molecular ligation with T4 DNA ligase. Primary bone marrow-derived macrophages from female A/J mice were isolated as described previously [88], frozen in 10% DMSO/90% fetal bovine serum, and thawed prior to use. Challenge of both primary A/J mouse bone marrow-derived macrophages and *Acanthamoeba castellanii* with *L. pneumophila* was performed as previously described [89], using bacteria grown overnight to post-exponential phase that were predominantly motile (A600 = 3.7–4.5). For growth curve analysis, 1×10^5^ macrophages per well were plated in 240 µl RPMI1640 without Phenol Red+glutamine+10% heat-inactivated FBS in 96-well white tissue culture treated plates (Greiner). *A. castellanii* (ATCC30234; American Type Culture Collection) were plated at a density of 2.5×10^5^ amoebae per well in 240 µl Ac buffer [89]. *H. vermiformis* (ATCC50237; American Type Culture Collection) were plated at a density of 1×10^5^ amoebae per well in 240 µl *H. vermiformis* medium (modified PYNFH medium, ATCC medium 1034). Macrophages and *A. castellanii* were challenged with bacteria at an MOI of 0.05; *H. vermiformis* were challenged at an MOI of 0.5. The plates were incubated at 37°C (in 5% CO_2_ for macrophages) in a Tecan Infinite M200 Pro with Luminescent and CO_2_ Gas Modules. Luminescence was measured for 20 seconds per well every 20 minutes. Growth experiments were performed multiple times, and in each case the data shown are from one representative experiment with 3 or more inoculations each experiment.

### Extended passage of *L. pneumophila* in macrophage culture

An overnight culture of LP01::*lux* bacteria was grown to post-exponential phase, as described above. 5×10^5^ bacteria were used to inoculate each of 4 independent cultures of 1×10^7^ freshly thawed A/J primary bone marrow-derived macrophages in 12 ml of RPMI1640+glutamine+10% heat-inactivated FBS+100 µg/ml streptomycin in 10 cm tissue culture treated Petri dishes. After inoculation, each dish was centrifuged for 5 minutes at 400× g and incubated at 37°C, 5% CO_2_ for 2–3 days. After incubation, supernatants from each culture were collected. Remaining host cells were lysed by adding 8 ml of sterile ultrapure water (Invitrogen) and incubating for 15 minutes at room temperature. After pipetting up and down, these lysates were combined with the supernatants. An estimate of bacterial density was determined by pelleting 1.5 ml of each collection, resuspending in 100 µl PBS, and measuring luminescence in a 96 well plate in a Molecular Devices Spectramax M5 plate reader. These estimates were empirically determined by using as standards the luminescence of single passages of wild type bacteria harvested in this manner after 3 days in culture. An amount of culture equal to approximately 1×10^−3^ of the growth over these 3 days was used to inoculate new cultures of 1×10^7^ macrophages thawed 1 day prior to this re-infection. Periodically, dilutions of these lysates were plated on solid CYE agar plates in order to ensure that luminescence continued to approximate the CFUs in each culture. Every 9–25 days, the remaining lysates were centrifuged for 5 minutes at 400× g to remove cellular debris and intact host cells. The supernatants were then centrifuged at 7000× g for 15 minutes and the resulting bacterial pellets were resuspended in AYE+20% glycerol and stored at −80°C for future analysis.

### Competition assays

For competition assays, host cells were challenged at total MOI = 0.05, consisting of equal mixtures of two strains, one carrying the *luxCDABE* operon and one without, centrifuged for 5 minutes at 400× g and incubated at 37°C, 5% CO_2_. These assays were performed in 96 well plates, using either 1×10^5^ primary A/J bone marrow-derived macrophages or 5×10^5^ *A. castellanii* cells. *A. castellanii* challenge was performed without centrifugation. 3 days after inoculation, remaining host cells were lysed with 0.05% saponin for 5–10 minutes as described previously [89]. Dilutions of each lysate were plated on CYE solid agar and colonies were visualized with and without epi-illumination using the Biorad Chemidoc XRS system. The competitive index (C.I.) was determined as described previously [31], C.I. = (mutant/wild type output ratio)/(mutant/wild type input ratio), normalized to the results from competitions between two differentially marked progenitor strains, and plotted on a logarithmic scale. P-values were determined in a two-tailed, unpaired Student's t-test of the logarithmic-transformed normalized C.I. values, comparing each competition to a wild type/wild type control competition. A P-value of less than or equal to 0.05 was considered a significant difference from this control competition.

Uptake competition assays were performed with 1×10^5^ primary A/J bone marrow-derived macrophages added to each well of a 96 well tissue culture plate. After a 4 hr incubation at 37°C, 5% CO_2_, host cells were challenged with a total MOI = 1.0, consisting of equal mixtures of two strains, centrifuged for 5 minutes at 400× g and incubated at 37°C, 5% CO_2_ for 1 hr. 1 hr after inoculation, gentamicin was added to each well at the final concentration of 50 µg/ml and the cultures were incubated for an additional hr to kill extracellular bacteria. Each well was then washed 5 times with 200 µl phosphate-buffered saline, after which macrophages were lysed by incubating each well in 200 µl ultrapure water for 10–15 minutes at room temperature. Relative ratios of colony forming units (CFUs) for each strain in each well were calculated as above. Control wells of bacterial inoculations, in which no macrophages were present, were also performed under these conditions in order to confirm the anti-bacterial activity of gentamicin against extracellular bacteria, as indicated by the absence of CFUs from these wells. A competitive index of uptake was determined for each challenge, as described above.

### Illumina whole-genome sequencing and analysis

Bacteria were recovered from glycerol stocks by streaking onto CYE agar plates. Genomic DNA was isolated from individual clones grown to post-exponential phase, using the Qiagen DNeasy kit including the optional RNase digestion (Ambion RNase cocktail). 5 µg of genomic DNA was sheared by nebulization for 6 minutes at 35 psi (Invitrogen). Sheared DNA was purified on QIAquick spin columns (Qiagen), then treated with the End-IT DNA Repair kit (Epicentre). After spin-column purification, 3′ A-tailing was performed by incubating for 1 hour at room temperature with Exo-minus Klenow (New England Biolabs) and dATP. Samples were again purified using QIAquick columns, and custom, 4 nt 5′ barcoded, adapter sequences were ligated to each sample using the Fast-link ligation kit (Epicentre). Libraries were size-selected on 2% agarose gels, and fragments 400–450 nucleotides in length were purified using QIAquick columns. To enrich for properly ligated samples, approximately 4% of each library was amplified for 16 cycles using common primers, QIAquick purified, and then quantified using a Nanodrop spectrophotometer. Libraries were mixed at equal ratios and sequenced on an Illumina Genome Analyzer II. LP01::*lux*, d133A, d200A, d314A, d133B, d314B, d90C, d180C, d180C′, d45D, d90D, and d180D libraries were sequenced using single-end, 40 nt sequencing reactions. d346A and d346B libraries were sequenced using paired-end, 2×40 nt reactions. Multiplexed sequence data was sorted by 5′ barcode identity into individual libraries. *De novo* assembly was performed with Velvet [90]. CLC Genomics Workbench 3 and Maq were used to generate reference assemblies comparing each strain against the published *L. pneumophila* Philadelphia-1 (Genbank accession: NC_002942) and the LP01::*lux* assembly. Raw Illumina reads from each sequenced strain were deposited as Sanger-formatted FASTQ files in the Dryad Digital Repository (doi:10.5061/dryad.95mt02sb).

### Chemically defined media and *in vitro* growth analysis

Chemically defined, Modified Ristroph media (MRM) [44], [73] supporting *L. pneumophila in vitro* growth was used to determine the growth requirements of strains harboring lysine-biosynthetic mutations. Bacteria were grown overnight with shaking at 37°C to an A600 = 2.0 in AYE medium. For each strain, 2 tubes containing bacteria equivalent to 1 ml of A600 = 2.0 were pelleted at 14,000× g, washed once in 1 ml of MRM(−)lysine, and resuspended in 10 ml of either MRM(−)lysine or MRM(+)lysine. 240 µl of each of these suspensions was aliquoted into each of 3 wells of a 96 well plate. The OD of each well was measured every 12 minutes during incubation at 37°C with shaking in a Biotek plate reader.

### qEGAN analysis

Sterile pipette tips were used to scrape each of the frozen glycerol stocks collected during the experiment. These samples were resuspended in ultrapure water, pelleted in a microcentrifuge, and cleared supernatants were used as templates for analysis.

The procedure used for determining the proportion of each allele is Quantitative Exon Grouping Analysis (qEGAN). This is an extension of the procedure used in the laboratory for high throughput sequence analysis of exonic regions that has been used extensively for the analysis of BRCA1 and BRCA2 [38]. qEGAN is a heteroduplex technology based on conformation-specific gel electrophoresis (CSGE) [91], [92] and conformation-specific capillary electrophoresis (CSCE) [93], [94].

Briefly, an approximately 200 nt region containing each polymorphism was PCR amplified using flanking primers containing universal 5′ sequence tails. These products were fluorescently labeled during a secondary round of PCR amplification, using FAM-tagged universal primers. These fluorescently labeled amplicons were directly analyzed on an Applied Biosystems 3730xl capillary sequencer with a 50 cm Capillary Array, using non-denaturing POP Conformational Analysis Polymer and ROX labeled size standard (Life Technologies, Carlsbad, CA). The curve height of fluorescent signal from each reaction was normalized and the relative patterns analyzed using DAx Data Acquisition and Data Analysis software (Van Mierlo Software Consultancy, Eindhoven, NL).

In parallel, mixtures of genomic DNA from LP01 and each adapted strain were also used as templates for this analysis. By comparing the curves generated from each known mixture of wild type∶mutant genomic DNA, the ratio of each mutation was determined within the time-course glycerol stocks. These results were confirmed by phenotypic quantification and results from allele-specific quantitative PCR (data not shown).

## Supporting Information

Table S1 **Whole genome resequencing of several clones from each lineage.** Illumina reads were aligned by reference assembly to both the published *L. pneumophila* Philadelphia-1 genome and to contigs from the progenitor strain. These alignments were used to identify mutations present in each strain. Each individual mutation was observed in isolates marked with an asterisk. Strain names in italics represent time-points and clones chosen for sequencing in order to explain population behavior identified in Figure 2. Two isolates from day 180 of the C lineage were sequenced, day 180 and day 180′.
(PDF)
