# Supplementary material for: Experimental Evolution of Legionella pneumophila in Mouse Macrophages Leads to Strains with Altered Determinants of Environmental Survival
Source: PLoS Pathog. 2012 May 31;8(5):e1002731. doi: 10.1371/journal.ppat.1002731 (PMC3364954; doi:10.1371/journal.ppat.1002731)
Supplement: Table S1 — Whole genome resequencing of several clones from each lineage. Illumina reads were aligned by reference assembly to both the published L. pneumophila Philadelphia-1 genome and to contigs from the progenitor strain. These alignments were used to identify mutations present in each strain. Each individual mutation was observed in isolates marked with an asterisk. Strain names in italics represent time-points and clones chosen for sequencing in order to explain population behavior identified in Figure 2. Two isolates from day 180 of the C lineage were sequenced, day 180 and day 180′. (PDF) [file ppat.1002731.s001.pdf]

Table S1 - Whole genome resequencing of several clones from each lineage.

A lineage				Day			
**genomic location**	**nt change**	**overlapping gene(s)**	**aa change**	**133**	***200***	**314**	**346**
324022	G->T	*sdbA*	G157opal			*	*
375184	C->G	*rpoC*	L293V	*			
411562	G->A	*fur*	G84S		*		
1074316	A->G	*lpg0981*	V46A	*			
1754709-1754710	deletion (-2)	*lpg1586*	frameshift		*	*	*
1991160	C->A	*fleN*	D75Y	*	*	*	*
2909408	G->C	*oivA*	S606opal				*
3041723	deletion (-1)	*lphB/pacS*	intergenic			*	*
3273231	deletion (-1)	*gidB*	frameshift		*		

B lineage				Day		
**genomic location**	**nt change**	**overlapping gene(s)**	**aa change**	**133**	**314**	**346**
325402	deletion (-1)	*sdbA*	frameshift	*	*	*
395104	C->G	*rpoA*	I46M			*
930001	T->A	*fleQ*	H179Q			*
2577569-2577571	deletion (-3)	*uqpQ*	K99del		*	
2915886	G->T	*sidF*	G848ochre		*	
3041723	deletion (-1)	*lphB/pacS*	intergenic			*
3084284	T->A	*lqsS*	N336Y	*	*	*
3356904	deletion (-1)	*argD*	frameshift	*	*	*

C lineage				Day		
**genomic location**	**nt change**	**overlapping gene(s)**	**aa change**	**90**	**180**	***180'***
897851	G->T	*clpA*	R646M			*
960031-960032	insertion (TGC)	*dapE*	A346_T347insA			*
1680473	C->G	*lssE (cdgS19)*	synonymous		*	
1991063	A->G	*fleN*	V107A		*	
2910291	C->A	*oivA*	E313ochre	*	*	*

D lineage				Day		
**genomic location**	**nt change**	**overlapping gene(s)**	**aa change**	**45**	**90**	**180**
323591	deletion (-1)	*sdbA*	frameshift			*
1990881-1990883	deletion (-3)	*fleN*	V168del	*		
2026944	G->A	*lysC/A*	G813R		*	*
2317543	C->A	*dam/lpg2076*	intergenic			*
3262574	G->A	*metG*	synonymous		*	*

Illumina reads were aligned by reference assembly to both the published *L. pneumophila* Philadelphia-1 genome and to contigs from the progenitor strain. These alignments were used to identify mutations present in each strain. Each individual mutation was observed in isolates marked with an asterisk. Strain names in italics represent time-points and clones chosen for sequencing in order to explain population behavior identified in Figure 2. Two isolates from day 180 of the C lineage were sequenced, day 180 and day 180'.
